# Structural Elucidation and Antioxidant Activities of Proanthocyanidins from Chinese Bayberry (*Myrica rubra* Sieb. et Zucc.) Leaves

**DOI:** 10.1371/journal.pone.0096162

**Published:** 2014-05-07

**Authors:** Yu Fu, Liping Qiao, Yuming Cao, Xiaozhou Zhou, Yu Liu, Xingqian Ye

**Affiliations:** 1 Department of Food Science and Nutrition, College of Biosystems Engineering and Food Science, Fuli Institute of Food Science, Zhejiang Key Laboratory for Agro-Food Processing, Zhejiang R & D Center for Food Technology and Equipment, Zhejiang University, Hangzhou, PR China; 2 College of Life Science, Zhejiang University, Hangzhou, China; National Cancer Institute at Frederick, United States of America

## Abstract

Proanthocyanidins in Chinese bayberry leaves (PCBLs) were qualitatively analyzed. NMR data suggest that PCBLs are mostly composed of (epi)gallocatechin gallate units. Matrix-assisted laser desorption time-of-flight MS data indicate 95 possible prodelphinidin structures, ranging from dimers to tridecamers. Preparative normal-phase HPLC and further analysis by reverse-phase HPLC together with electrospray ionization MS enabled detection of 20 compounds, including seven newly identified compounds in Chinese bayberry leaves. The antioxidant capacity of PCBLs was evaluated by (1,1-diphenyl-2-picryl-hydrazyl), ferric-reducing antioxidant power, and oxygen radical absorption capacity assays. The EC_50_ of DPPH radical scavenging activities (as 50% decrease in the initial DPPH concentration) were 7.60 µg. The FRAP and ORAC values were 8859.33±978.39 and 12991.61±1553.34 µmol Trolox equivalents per gram, respectively. The results indicate the high antioxidant potency of PCBLs.

## Introduction

**Figure 1:**
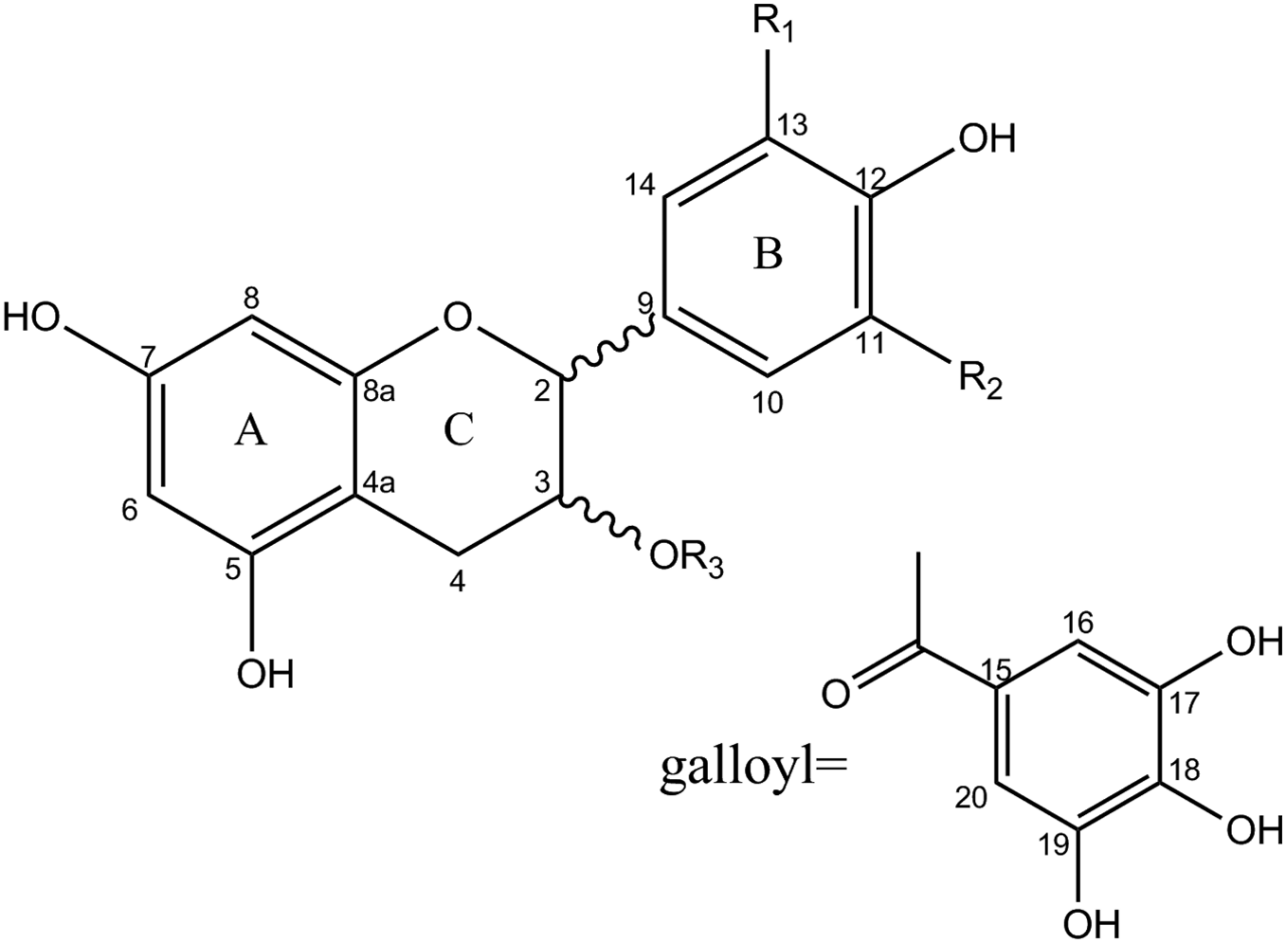
Basic structure of proanthocyanidins. R1, R2, R3 = H, propelargonidins; R1, R3 = H, R2 = OH, procyanidins; R1, R2 = OH, R3 = H, prodelphinidins; R3 = galloyl, galloylated proanthocyanidins.

Proanthocyanidins (PAs), also called condensed tannins, are a class of colorless phenolics characterized by an oligomeric or polymeric structure based on flavan-3-ol units [1]. PAs can be divided into several classes on the basis of the hydroxylation patterns of their constitutive units and the linkages between them (Figure 1). Flavan-3-ol units are most frequently linked via B-type bonds, namely, C4→C8 or C4→C6 linkages [2]. Occasionally, an additional C2→O7 or C2→O5 linkage may exist, leading to doubly bonded A-type PAs [3]. Polymeric PAs can be classified as B-type PAs, which are linked mainly with B-type linkages and A-type PAs, which have more abundant A-type bonds as well as B-type linkages [4]. The most common constitutive units of propelargonidin, procyanidin, and prodelphinidin are (epi)afzelechin [(E)AF], (epi)catechin [(E)CA], and (epi)gallocatechin [(E)GC], respectively [3]. Some of these units could also be esterified with other moleculars such as glucose or gallic acid[5]. The chromatographic separation of PAs is complicated because of the enormous variety of similar isomeric oligomers in plant or food sources [6]. PAs are used for treating periodontal diseases [7]. They have also been reported to demonstrate antioxidant [8], antimicrobial [9], [10], anti-diabetic [11], anti-angiogenic [12], anticarcinogenic [13], anti-inflammatory [14] and antimelanogenic [14] activities.

Chinese bayberry (*Myrica rubra* Sieb. et Zucc., Myricaceae) is a subtropical evergreen fruit tree widely grown in Southern China [15], [16]. Most of its cultivars in China blossom in March and April, and then the fruit ripens in June and July [15]. In the book of Chinese herbal medicine, *Compendium of Materia Medica*, medical application of the leaf cures enteritis and arthralgia and promotes hemostasis [17]. Recent research has mainly focused on the function of myricetin isolated from *M. rubra* Sieb. et Zucc. leaves, including analgesic [18] and anti-inflammatory activities [19]. Research on PAs of *M. rubra* Sieb. et Zucc. leaves are limited. Masuda et al. identified two monomers (EGC and EGCG)[20]; Yang et al. identified five flavan-3-ol monomers (EGCG) and oligomeric PAs (EGC-EGCG, 2EGCG, 2EGCG+EGC and 3EGCG), and elucidated the structural features of bayberry leaf PAs by using acidic degradation [21].

The main objective of this work was to characterize the structures of PAs extracted from Chinese bayberry leaves. This was achieved by fractionation on normal-phase HPLC at preparative scale to enable separation of PAs according to their polymerization degrees. Further fractionation facilitated their analysis by reversed-phase HPLC coupled to UV–visible (UV–vis) spectroscopy and electrospray ionization mass spectrometry (ESI/MS). The combination of NP- and RP-HPLC to identify proanthocyanidins has rarely been used. We also attempted to use matrix-assisted laser desorption time-of-flight mass spectrometry (MALDI-TOF MS) and NMR spectroscopy to obtain more information about polymers and evaluate the antioxidant potential of PCBLs.

## Materials and Methods

### 2.1 Ethics Statement

We contact a local farmer who owned a field of bayberry trees in Cixi and harvested the leaves under his permission. In this case, no conflicts of interests are existed and no endangered or protected species are included. The specific location is around 30.075315, 121.562494.

### 2.2 Materials

Leaves of *Biqi*, the major cultivar used in the industry, were hand-harvested randomly in October 29, 2011 in Cixi, Zhejiang Province, Southeastern China. Sephadex LH-20 was purchased from GE Healthcare Bio-Sciences AB (Sweden). Methanol, hexane, and ethyl acetate for HPLC analysis and preparation were of HPLC grade. All other reagents and solvents used were of analytical grade, unless stated otherwise. 2,5-Dihydroxybenzoic acid (DHB), trifluoroacetic acid (TFA), 6-hydroxy-2,5,7,8-tetramethyl-choman-2-carboxylic acid (Trolox), 2,2′-azobis(2-methylpropionamidine)dihydrochloride (AAPH), (1,1-diphenyl-2-picryl-hydrazyl) (DPPH), 2,4,6-tri(2-pyridyl)-1,3,5-triazine (TPTZ), 2′,7′-dichlorofluorescin diacetate, and fluorescein disodium salt were purchased from Sigma-Aldrich (St. Louis, MO, USA). Myricetin was purchased from J&K Scientific Ltd. (Beijing, China). Sodium chloride and L-ascorbic acid were purchased from Sinopharm Chemical Reagent Co., Ltd. (Shanghai, China). Gallocatechin (GC), epicatechin gallate (ECG), gallocatechin gallate (GCG), and epigallocatechin gallate (EGCG) were purchased from Yuanye Bio-Technology Co., Ltd. (Shanghai, China).

### 2.3 Extraction and Purification of PAs

Extraction and purification of PAs from Chinese bayberry leaves were carried out essentially according to our previous procedures [21]. In brief, the leaves were dried at 40°C for 12 h and then ground well into a powder by milling. The finely ground powder (4 kg) was extracted with 70% aqueous acetone (40 L) containing 0.1% (w/v) ascorbic acid at room temperature for 12 h. The extraction was performed two times. The acetone extracts were pooled and rotary-evaporated under vacuum at 40°C to remove acetone. The aqueous phase (∼20 L) was recovered and washed with hexane (2×20 L) to remove nonpolar material, and then the organic solvents were evaporated under vacuum. Finally, the aqueous phase was lyophilized to dryness to obtain the bayberry leaf extracts (BLEs, ∼400 g).

Adsorption chromatography on Sephadex LH-20 has been proven to be very suitable for sample cleanup and fractionation of PAs according to their molecular weight [22]. To purify the BLEs, a solution of 4 g of the extracts redissolved in 5 mL of 50% methanol was loaded onto a column (300 mm×30 mm i.d.) of Sephadex LH-20, and eluted stepwise with 300 mL of 50% methanol to elute pigments and sugars, 300 mL of 90% methanol to remove most flavonoids, 300 mL of 50% acetone to remove most PAs, and then 300 mL of 70% acetone to clean the column. Consecutive fractions collected in 300 mL portions were labeled fractions 1 (2.06±0.11 g), 2 (0.45±0.02 g), 3 (0.88±0.08 g), and 4 (0.52±0.03 g) (*n* = 10), rotary-evaporated under vacuum to remove the organic solvents, and lyophilized to dryness. Fraction 3 (PCBLs) was used for further analysis.

### 2.4 NMR Spectroscopy

^13^C NMR spectra of the sample (50 mg) were recorded on a Bruker Avance III 600 spectrometer (Bruker BioSpin Inc., Fällanden, Switzerland), using CD_3_OD (Cambridge Isotope Laboratories, Inc., U.S.A.) as the solvent (0.6 mL) and tetramethylsilane as the internal standard (0.00 ppm). GC, ECG, GCG, and EGCG were used as standards.

### 2.5 MALDI-TOF MS

PCBLs were further analyzed by MALDI-TOF MS. The matrix solution was prepared by dissolving DHB (10 mg) and sodium chloride (cationization reagent, 1 mg) in aqueous 1% aqueous TFA (1 mL). Aliquots of sample and matrix solutions were mixed (1∶1, v:v), vortexed, and then deposited (2 µL) on a stainless steel metal plate. Once the solvent was dried (at room temperature), the crystals were analyzed using an ABI Voyager DE-STR instrument in positive-ion reflectron mode.

### 2.6 Normal-phase HPLC–ESI/MS Analysis and Preparation

Analysis was performed on a Waters platform, composed of a Waters 2695 HPLC unit equipped with a Waters 2998 photodiode array (PDA) detector and a Luna silica column (4.6 mm i.d. ×250 mm, 5 µm particle size; Phenomenex, Torrance, CA, USA). The unit was coupled with a Bruker Esquire 3000 Plus ion trap mass spectrometer (Bruker–Franzen Analytik GmbH, Bremen, Germany) equipped with ESI. The PCBLs was dissolved in methanol to prepare a 20 mg/mL solution. The mobile phases consisted of hexane/methanol/ethyl acetate (10∶3∶1, v/v/v) (A) and hexane/methanol/ethyl acetate (1∶3∶1, v/v/v) (B). Separations were done by linear gradient at 37°C at 1 mL/min flow rate as follows: 0–90 min, 83.6% A; 90–130 min, 83.6–19.4% A; 130–150 min, 19.4% A. The PDA detector was set to 280 nm and scanning was done from 200 to 400 nm.

Preparation was performed on a Phenomenex (Torrance, CA, USA) Luna silica preparative column (21.2 mm i.d. ×250 mm) with a 5 µm particle size at 37°C. A Shimadzu system equipped with a CBM-20A module, an SIL-10AP autosampler, an SPD-20A UV–vis detector, and two LC-8A pumps was used. Elution was the same as the method described above. The flow rate was 21.6 mL/min and the absorption wavelength was at 280 nm. On a given run, 1 mL (200 mg/mL) extract was applied. The fractions were collected and evaporated under vacuum.

### 2.7 Reversed-phase HPLC–ESI/MS Analysis

A Shimadzu system equipped with a CBM-20A module, an SIL-20A autosampler, an SPD-M20A diode array detector, an LC-20AB pump, a CTO-20A column oven, and an LCMS2020 mass detector was used. Analysis was performed on a Zorbax SB-C18 column (4.6 mm i.d. ×250 mm, 5 µm particle size; Agilent, USA). Fractions collected from normal-phase preparative HPLC were dissolved in methanol. The mobile phase was composed of H_2_O–0.1% (v/v) acetic acid (A) and methanol (B). The flow rate was set at 1 mL/min and detection was performed at 280 nm. The elution was done as follows. First 10 min, isocratic with 80% A; 10–40 min, linear decrease of 80–50% A; 40–50 min, linear decrease of 50–10% A; 50–55 min, linear increase of 10–80% A; 55–60 min, isocratic with 80% A.

### 2.8 Antioxidant Activity

#### DPPH assay

The DPPH assay was performed as previously described [23], with slight modification. In brief, 3.9 mL of 0.1 mmol/L DPPH in ethanol was added to 0.1 mL sample extracts. The resulting sample mixtures were stored at room temperature in the dark conditions and their absorbance was taken at 517 nm. The result was expressed as EC_50_.

#### Ferric-reducing capacity assay

The ferric-reducing antioxidant power (FRAP) assay was determined according to a previous report [24], with slight modification. The FRAP reagent consisted of 0.1 mol/L acetate buffer (pH 3.6), 10 mmol/L TPTZ (dissolved in 40 mmol/L HCl), and 20 mmol/L ferric chloride at a ratio of 10∶1∶1 (v/v/v). Samples (100 µL) were allowed to react with 3.9 mL of prepared FRAP working solution for 10 min at 37°C in the dark, and then the absorbance was measured at 593 nm. The results were expressed as the number of micromoles of Trolox equivalents per gram of PCBLs.

#### Oxygen Radical Absorption Capacity (ORAC) assay

The ORAC were determined using a Fluoroskan Ascent FL plate-reader (Thermo Fisher Scientific, USA) with 96-well plates [25]. The fluorescein solution was prepared with 75 mmol/L potassium phosphate buffer (pH 7.4) to a final concentration of 504 nmol/L. Subsequently, 25 µL of sample/Trolox solution and 25 µL of fluorescein solution was added to a black bottom 96-well plate, which was then placed in a water bath at 37°C for 5 min. Afterward, 150 µL of 17.07 mmol/L AAPH potassium phosphate buffer solution was added to the mixture. After shaking for 10 s, absorption measurements were recorded every 1 min for 2 h at 37°C. Emission and excitation wavelengths were 565 and 540 nm, respectively. ORAC values were expressed as the number of micromoles of Trolox equivalents per gram of PCBLs.

### 2.9 Statistical Analysis

All samples tested in antioxidant activities were prepared and analyzed in triplicate. The values of means ± SD were calculated. The 50% decrease in the initial DPPH concentration (EC50), under the experimental conditions, was obtained by linear regression analysis of the inhibition plots (Origin 8.0 software).

## Results and Discussion

### 3.1 Identification of PAs by NMR

**Figure 2:**
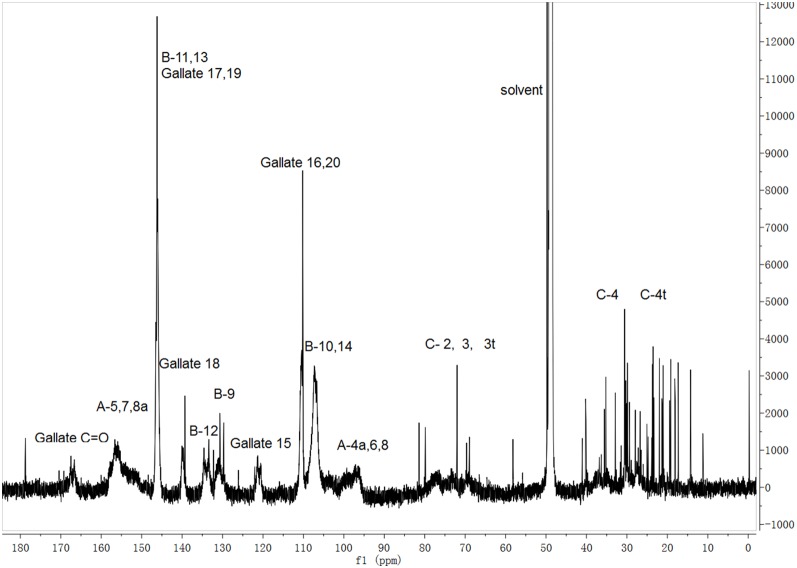
^13^C NMR spectrum of Chinese bayberry leafs proanthocyanidins. Samples were dissolved in methanol-*d*_4_.

A previous study based on thiolytic degradation with phloroglucinol has shown that PCBLs are essentially of the prodelphinidin type, and more than 98% of them were galloylated [21]. The ^13^C NMR spectrum of the crude bayberry PAs (Figure 2) has the characteristic features of a mixed PA with 3-O-galloylation on some subunits [26]. Resonances of the gallate carbonyl (166 ppm) and galloyl ring carbons C18 and C16/C20 (138 and 110 ppm, respectively) are unique to galloylated flavan-3-ols [27]. The characteristic peaks for the A-ring carbons (∼95, ∼155 ppm) and B-ring carbons (∼108, 121, 133, 145 ppm) are consistent with a prodelphinidin polymer, but do not provide specific structural detail [28]. No carbon signals were observed between δ 110 and 120, which correspond to B-ring C10/C13 carbon in procyanidin or B-ring C11/C13 carbon in propelargonidin. Furthermore, the low solubility in methanol and acetone, as well as the carbon signals between δ 60 and 82 may indicate the presence of carbohydrate moieties in the fraction [29]. NMR studies of the standards and PCBLs indicated that most of the bayberry leaf PAs were composed of (E)GCG units (Table 1), which are consistent with the thiolytic degradation result [21].

**Table 1:** ^13^C (600 MHz) NMR Data of GC, ECG, GCG, EGCG in Methanol-d4.

position	GC	ECG	GCG	EGCG
C-2	81.4	77.2	77.8	77.2
C-3	67.4	68.6	69.7	68.6
C-4	26.7	25.5	22.3	25.4
C-4a	99.3	98.0	98.2	98.1
C-5	155.4	155.9	155.0	155.8
C-6	94.9	95.2	95.0	95.2
C-7	156.4	156.5	156.7	156.5
C-8	94.1	94.5	94.2	94.5
C-8a	156.2	156.4	156.2	156.4
C-9	130.2	130.1	129.6	129.4
C-10	105.8	113.7	104.9	105.5
C-11	145.5	144.6	145.6	145.3
C-12	132.6	144.6	132.6	132.4
C-13	145.5	114.6	145.6	145.3
C-14	105.8	118.0	104.9	105.5
C-15		120.1	120.0	120.1
C-16		108.8	108.8	108.9
C-17		144.9	145.0	144.9
C-18		138.4	138.5	138.4
C-19		144.9	145.0	144.9
C-20		108.8	108.8	108.9
C = O		166.2	166.3	166.3

### 3.2 Identification of PAs by MALDI-TOF MS

Analyses performed using LC–ESI/MS have shown that several oligomeric structures of PCBLs mainly consist of EGCG units with a high proportion of B-type structures [21]. However, there is little information about highly polymerized PAs. In order to obtain more structural information on PCBLs, MALDI-TOF MS was first used to analyze the purified fraction of Chinese bayberry leaves.

The theoretical monoisotopic mass (as sodium adducts, [M+Na]^+^) corresponding to prodelphinidins may be calculated as follows: [M+Na]^+^ = 306.07×GC+152.01×GALLOYL−2.02×*B*−4.04×*A*+22.99, where GC is the number of (E)GC units contained in the PA molecule, GALLOYL is the number of galloyl ester units attached to the flavan-3-ol units; and *B* and *A* are, respectively, the numbers of B- and A-type linkages between units.

Table 2 shows the calculated and observed values for Na adducts of PAs from dimers to tridecamers containing possible combinations of GC, galloyl residues, and number of A-type linkages. There are 40 mass values related to prodelphinidins observed by MALDI-TOF MS. As the degree of polymerization increased, the number of possible structures of one mass value also increased. Ninety-five possible compounds are listed in Table 2.

**Table 2:** Calculated and Observed Mass Values for Na Adduct Ions of PDs Dimers to Tetradecamers in PCBLs by MALDI-TOF/MS.

DP	Obs	Cal	Gal	N_A_ a	DP	Obs	Cal	Gal	N_A_ a	DP	Obs	Cal	Gal	N_A_ a	DP	Obs	Cal	Gal	N_A_ a
2	633.45	633.11	0	0	6	1836.95	1837.19	0	5	8	3671.70	3671.47	8	0	12	4103.69	4103.38	3	12
2	783.15	783.10	1	1	6	1990.87	1991.22	1	4	9	2745.43	2745.3	0	7	12	4265.37	4265.49	4	7
2	784.86	785.12	1	0	6	1993.15	1993.24	1	3	9	2759.49	2759.44	0	0	12	4719.48	4719.5	7	8
2	935.40	935.11	2	1	6	1997.02	1997.28	1	1	9	2897.33	2897.31	1	7	12	4869.36	4869.49	8	9
2	937.35	937.13	2	0	6	2147.66	2147.27	2	2	9	3213.27	3213.45	3	1	12	4879.46	4879.59	8	4
3	937.35	937.16	0	0	6	2293.01	2293.22	3	5	9	3517.86	3517.47	5	1	12	5037.42	5037.66	9	1
3	1084.92	1085.13	1	2	6	2300.83	2301.3	3	1	9	3671.70	3671.5	6	0	13	3949.02	3949.38	0	13
3	1239.43	1239.16	2	1	6	2597.32	2597.24	5	5	9	3810.95	3811.39	7	6	13	4103.69	4103.41	1	12
3	1391.38	1391.17	3	1	6	2759.49	2759.35	6	0	9	3815.20	3815.43	7	4	13	4265.37	4265.52	2	7
4	1239.43	1239.19	0	1	7	2147.66	2147.3	0	2	10	3213.27	3213.48	1	1	13	4719.48	4719.53	5	8
4	1391.38	1391.2	1	1	7	2293.01	2293.25	1	5	10	3517.86	3517.5	3	1	13	4869.36	4869.52	6	9
4	1691.37	1691.18	3	3	7	2593.66	2593.23	3	7	10	3671.70	3671.53	4	0	13	4879.46	4879.62	6	4
4	1692.74	1693.2	3	2	7	2597.32	2597.27	3	5	10	3810.95	3811.42	5	6	13	5037.42	5037.69	7	1
4	1697.04	1697.24	3	0	7	2745.43	2745.24	4	7	10	3815.20	3815.46	5	4	13	5642.14	5641.69	11	3
4	1848.95	1849.25	4	0	7	2759.49	2759.38	4	0	10	4265.37	4265.43	8	7	14	4265.37	4265.55	0	7
5	1691.37	1691.21	1	3	7	2897.33	2897.25	5	7	11	3517.86	3517.53	1	1	14	4719.48	4719.56	3	8
5	1692.74	1693.23	1	2	7	3213.27	3213.39	7	1	11	3671.70	3671.56	2	0	14	4869.36	4869.55	4	9
5	1697.04	1697.27	1	0	8	2439.70	2439.23	0	8	11	3815.2	3815.49	3	4	14	4879.46	4879.65	4	4
5	1848.95	1849.28	2	0	8	2593.66	2593.26	1	7	11	4265.37	4265.46	6	7	14	5037.42	5037.72	5	1
5	1993.15	1993.21	3	4	8	2597.32	2597.3	1	5	11	4719.48	4719.47	9	8	14	5642.14	5641.72	9	3
5	1997.02	1997.25	3	2	8	2745.43	2745.27	2	7	11	4869.36	4869.46	10	9					
5	2147.66	2147.24	4	3	8	2759.49	2759.41	2	0	11	4879.46	4879.56	10	4					
5	2152.84	2153.3	4	0	8	2897.33	2897.28	3	7	11	5037.42	5037.63	11	1					
5	2300.83	2301.27	5	2	8	3213.27	3213.42	5	1	12	3671.7	3671.59	0	0					
6	1835.22	1835.17	0	6	8	3517.86	3517.44	7	1	12	3815.2	3815.52	1	4					

a N_A_, number of A-type linkage.

### 3.3 Identification of Oligomeric PAs by Normal-phase HPLC–ESI/MS

Normal-phase HPLC using a bare silica column and elution with an organic mobile phase do not allow isolation of individual compounds, but they can separate PAs with the same degree of polymerization [6]. Normal-phase chromatography using a dichloromethane/methanol/formic acid/water mixture was used for the separation of PAs in grape seeds, grape skin, Saskatoon berries, almond skins, cocoa, chocolate, peanut skins, and blueberries [3], [30], [31], [32], [33], [34]. However, this method is unsuitable for large-scale fractionation because it utilizes a chlorinated solvent and a strong acid, which raise concerns on laboratory exposure, environmental protection, and disposal costs. Shoji et al. presented a modified method to achieve the separation of apple procyanidin polymers by normal-phase chromatography using a hexane/methanol/ethyl acetate mixture as the mobile phase [35].

**Figure 3:**
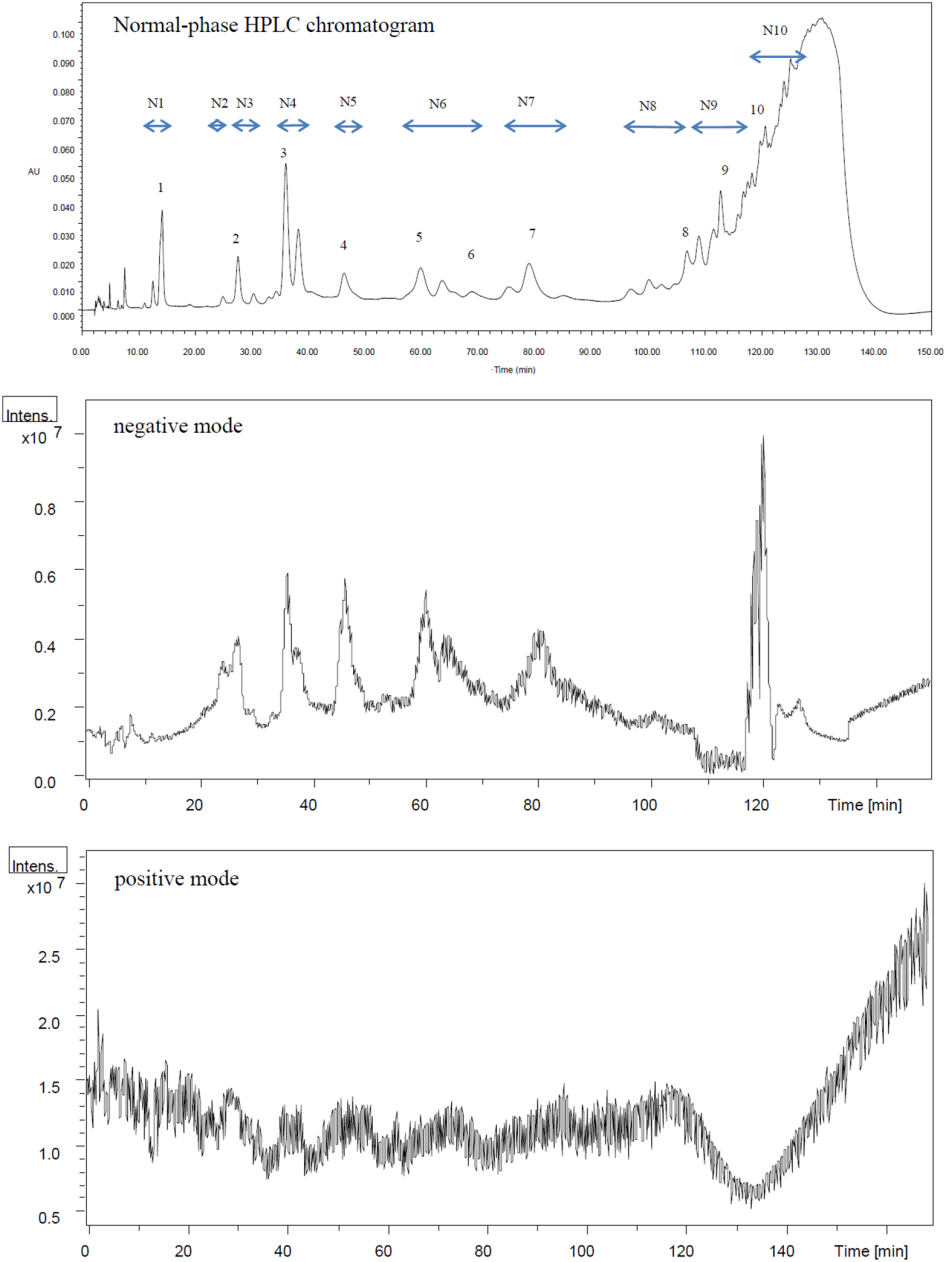
Normal-phase HPLC chromatograms and negative and positive ESI-MS spectra of Chinese bayberry leaf proanthocyanidins. N1 to N10 referred to fractions collected from preparative normal-phase HPLC according to the collecting time in Table 3.

Separation of PCBLs according to their polymerization degrees could be achieved to a certain extent on normal-phase HPLC using a hexane/methanol/ethyl acetate solution as the mobile phase (Figure 3). The crude extracts were run in both positive and negative ESI. As we can see in Figure 3, the negative mode provided much better sensitivity, which was in accordance with a previous report [36]. The detailed mass information of the peaks in normal-phase chromatography is given in Table 3. Compounds were tentatively identified on the basis of their retention time, UV spectra, and MS patterns; data in related literature were also taken into account.

**Table 3:** Fractionation information by normal-phase preparative HPLC and tentative compound identification by normal-phase HPLC-ESI/MS in negative mode.

NP^a^	CT^b^ (min)	Yield^c^ (mg/200 mg)	ID^d^	t_R_ e (min)	λ_max_ (nm)	N_A_ f	MW^g^	prominent ions, m/z	tentative identification^h^
N1	10–15.5	10.8±03	1	11.6	256, 349	-	616	615.4, 317.3	myricetin deoxyhexoside-gallate
N2	24–25.8	4.6±0.1	-	-	-	-	-	-	-
N3	26–31.5	8.6±0.3	2	26.9	274	0	762	761.3, 591.3	(E)GC+(E)GCG
N4	35–41	8.6±0.4	3	35.5	276	0	914	913.5, 743.2, 423.4	2(E)GCG
N5	46–50	4.3±0.2	4	45.6	274	0	1066	1163.3, 1065.6, 423.4	2(E)GC+(E)GCG
N6	57–72	8.7±0.2	5	60.1	276	0	1218	1315.3, 1217.7, 423.3	(E)GC+2(E)GCG
			6	69.5	276	1	1369	1367.5, 1217.4, 1005.5, 892.1	3(E)GCG, 3(E)GC+(E)GCG
N7	74–88	13.0±1.5	7	79.9	275	0	1371	1467.3, 1369.8, 423.4	3(E)GCG, 3(E)GC+(E)GCG
N8	97–108	19.6±0.6	8	106.9	276	0	1523	1522.0, 1369.3	2(E)GC+2(E)GCG
N9	108–118	32.2±1.6	9	114.6	276	0	1675	1674.0, 1521.6, 1369.5	(E)GC+3(E)GCG
N10	118–128	43.8±1.0	10	120.6	276	0	1827	1825.7, 1673.4, 1521.4, 1369.3, 1217.2, 991.5	3(E)CG+(E)GCG, 4(E)GCG

a NP, fraction numbers.

b CT, collecting time period.

c Yield, yield of one injection of preparative HPLC, that is, milligram per 200 milligrams bayberry leaf proanthocyanidin extract (BLPEs).

d ID, peak numbers.

e t_R_, retention time.

f N_A_, number of A-type linkage.

g MW, molecular weight.

h (E)GC, (E)GCG, (E)CG are abbreviations for (epi)gallocatechin, (epi)gallocatechin-3-O-gallate, (epi)catechin-3-O-gallate.

Peak 1 in fraction N1 apparently indicates flavonols since two major absorption peaks (256, 349 nm) are present in the UV–vis spectra. It represents a molecular ion [M–H]^−^ at *m/z* 615.4 with a fragment ion at *m/z* 317.3, which is a typical mass in the negative mode of myricetin aglycone. The MS spectra indicate the existence of a galloyl group (152) and a deoxyhexosyl group (615.3−152−317.3 = 146). Thus, compound **1** was tentatively identified as myricetin deoxyhexoside gallate, which is consistent with explanations in the literature [37].

**Figure 4:**
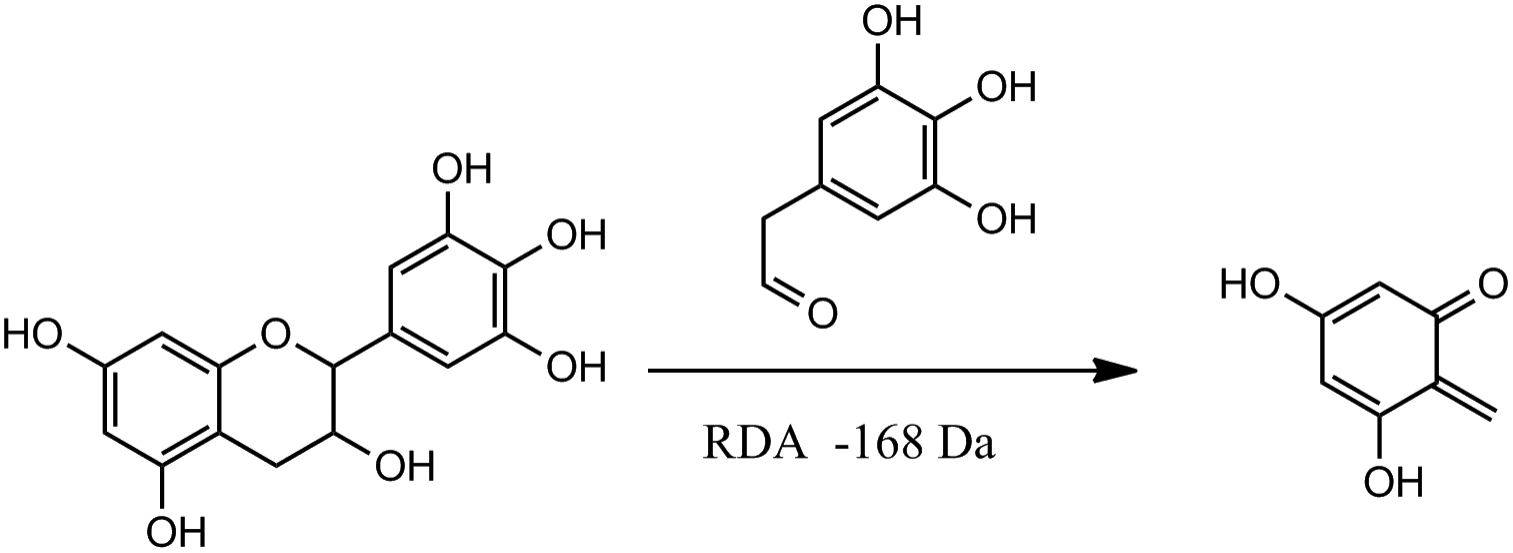
Retro-Diels–Alder (RDA) fragmentation pattern of gallocatechin.

The absorption maxima of peaks **2**–**10** at 274–276 nm appear to belong to PAs. The characteristic fragmentation pathways for PAs are the quinone methide mechanism, heterocyclic ring fission, and retro-Diels–Alder (RDA) fission, as previously described [38], [39], [40]. RDA fragmentation gives information about hydroxylation of the B rings and bonds between the two monomeric units. For example, neutral losses through RDA fissions from (E)AF, (E)CA, and (E)GC units are 136, 152, and 168 Da (Figure 4), respectively [40].

Compound **2** was tentatively identified as a gallate of a B-type dimeric (E)GC. It produced a pseudomolecular ion [M–H]^−^ at *m/z* 761.3 with a fragment ion at *m/z* 591.3 by the loss of gallic acid [21], [39].

Compound **3** could be a B-type dimer consisting of two (epi)gallocatechin-3-*O*-gallate [(E)GCG] units or a B-type trimer consisting of three (E)GC units. This compound produced a pseudomolecular ion [M–H]^−^ at *m/z* 913.5 with two fragment ions at *m/z* 743.2 by the loss of gallic acid, and at *m/z* 423.4 ([M–H^+^-170 Da-152 Da-168 Da]^−^) by the loss of two gallic acid fragments and an RDA fragment. According to the fragmentation information, this compound was assigned as a B-type dimer consisting of two (E)GCG units.

Compound **4** was tentatively assigned as a B-type trimer consisting of two (E)GC units and one (E)GCG unit. This compound produced a pseudomolecular ion [M–H]^−^ at *m/z* 1065.6 with a fragment ion at *m/z* 423.4 ([M–H^+^-170 Da-152 Da-152 Da-168 Da]^−^) by the loss of one gallic acid and three RDA fragments.

Compound **5** was tentatively assigned as a B-type trimer consisting of one (E)GC unit and two (E)GCG units. This compound produced a pseudomolecular ion [M–H]^−^ at *m/z* 1217.7 with a fragment ion at *m/z* 423.4 ([M–H^+^-170 Da-152 Da-152 Da-152 Da-168 Da]^−^) by the loss of two gallic acid and three RDA fragment.

Compounds **6** and **7** were tentatively assigned to a tetramer consisting of three (E)GC units and one (E)GCG unit or a trimer consisting of three (E)GCG units. According to the mass information, **6** seems to have resulted from the loss of two hydrogens of **7**, which fits the molecular weight of B-type PAs. Thus, **6** was presumed to contain one A-type linkage.

Compound **8** was tentatively assigned as a B-type tetramer consisting of two (E)GC units and two (E)GCG units. This compound produced a pseudomolecular ion [M–H]^−^ at *m/z* 1522.0 with a fragment ion at *m/z* 1369.3 by the loss of a galloyl residue.

Compound **9** was tentatively assigned as a B-type tetramer consisting of one (E)GC units and three (E)GCG units. This compound produced a pseudomolecular ion [M–H]^−^ at *m/z* 1674.0 with two fragment ions at *m/z* 1521.6 by the loss of a galloyl residue, and at *m/z* 1369.5 by the further loss of a galloyl residue.

Compound **10** was tentatively assigned as a B-type tetramer consisting of three (E)CG units and one (E)GCG unit or four (E)GCG units. This compound produced a pseudomolecular ion [M–H]^−^ at *m/z* 1825.7 with fragment ions by the loss of a galloyl residue or RDA fragment.

This is the first known report of detection of tetrameric PAs and A-type PAs in Chinese bayberry leaves. A trimer consisting of two (E)GC units and one (E)GCG unit was also first identified. As the degree of polymerization increases, the fission pathway of the compound could have a large number of possibilities. More information is needed to confirm the structure of these compounds.

The mass information was used as a reference to set the collection time for normal-phase preparative liquid chromatography. Each fraction was collected manually and evaporated to give quantitative information, which was presented in Table 3. Fractions containing dimers and trimers (N2–N7) made up almost 5% of the BLEs, whereas fractions containing tetramers (N8–N10) composed almost 10% of the BLEs.

### 3.4 Identification of Oligomeric PAs by Reversed-phase HPLC–ESI/MS

**Figure 5:**
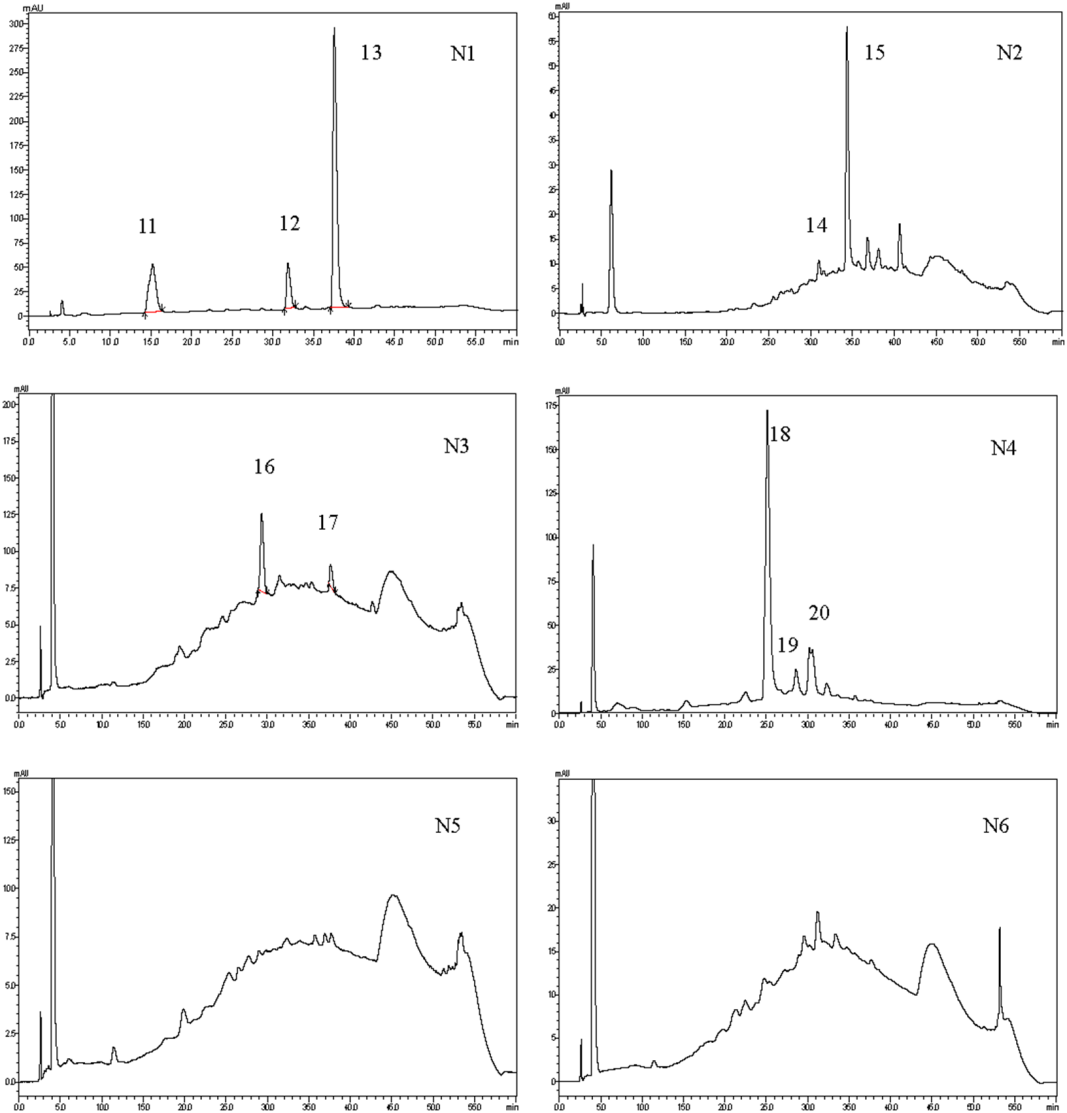
Reversed-phase HPLC chromatograms of fractions collected from preparative normal-phase HPLC. N1 to N6 referred to fractions collected from preparative normal-phase HPLC according to the collecting time in Table 3.

Results for each fraction analyzed by reversed-phase HPLC–ESI/MS are given in Figure 5 and Table 4.

**Table 4:** Tentative compound identification by reverse-phase HPLC-ESI/MS in both negative and positive mode.

NP^a^	ID^b^	t_R_ c (min)	λ_max_ (nm)	N_A_ d	MW^e^	negative mode	positive mode	Tentative identification^f^
N1	11	15.2	274	0	458	915.15, 571.10, 479.10, 457.05	939.20, 707.35, 561.10, 481.05, 459.10	EGCG
N1	12	31.9	257, 351	-	464	927.20, 531.10, 463.05	951.20, 716.50, 487.10, 465.10, 319.00	myricetin deoxyhexoside
N1	13	37.6	266, 351	-	616	1231.20, 615.10	944.55, 639.15, 617.15	myricetin deoxyhexoside-gallate
N2	14	31.1	275	1	744	1510.05, 743.15	767.10, 745.15, 481.30, 407.25	(E)GC+(E)CG
N2	15	34.4	268, 361	-	882	765.10, 631.05, 382.05	883.15, 789.15, 767.20, 719.30, 655.10, 633.05, 481.25, 368.20	2(E)CG, 2(E)C+(E)GC
N3	16	29.3	270, 349	-	882	765.15, 446.95, 360.10	882.80, 789.15, 767.10, 720.10, 655.20, 621.20, 481.25	2(E)CG, 2(E)C+(E)GC
N3	17	37.7	270, 349	-	616	615.1	639.50, 588.40, 566.50	myricetin deoxyhexoside-gallate
N4	18	25.3	275	1	1369	1367.90, 922.55, 911.50, 455.25	1391.55, 935.15, 913.15, 508.20, 492.10	3(E)GCG
N4	19	28.6	275	1	912	911.20, 489.20, 455.25	935.15, 913.15, 508.05, 333.15	2(E)GCG
N4	20	30.3	276	1	912	911.25, 489.25, 455.30	935.15, 913.15, 508.25	2(E)GCG

a NP, fraction numbers.

b ID, peak numbers.

c t_R_, retention time.

d N_A_, number of A-type linkage.

e MW, molecular weight.

f (E)GC, (E)GCG, (E)CG are abbreviations for (epi)gallocatechin, (epi)gallocatechin-3-O-gallate, (epi)catechin-3-O-gallate.

Dimers of the form [2M–H]^−^, [2M+Na–2H]^−^, [2M+H]^+^, and [2M+Na]^+^ were observed in compounds **11**–**14**. Compound **11**, which produced the pseudomolecular ion [M–H]^−^ at *m/z* 457.05 and [M+H]^+^ at *m/z* 459.10, was tentatively assigned as (E)GCG. This compound produced the fragment ions [2M–H]^−^ and [M+Na–2H]^−^ at *m/z* 915.15 and 479.10, respectively, in negative mode; as well as [2M+Na]^+^ and [M+Na]^+^ at *m/z* 939.20 and 481.05 in positive mode.

Compound **12** was tentatively assigned as myricetin deoxyhexoside. It produced the pseudomolecular ion [M–H]^−^ at *m/z* 463.05 and [M+H]^+^ at *m/z* 465.10. This compound produced the fragment ion [2M–H]^−^ at *m/z* 927.20 in negative mode, as well as the fragment ions [2M+Na]^+^ and [M+Na]^+^ at *m/z* 939.20 and 487.10, respectively, in positive mode.

Compound **13** was tentatively assigned as myricetin deoxyhexoside gallate, which produced the pseudomolecular ions [M–H]^−^ and [M+H]^+^ at *m/z* 615.10 and 617.15, respectively. This compound produced the fragment ion [2M–H]^−^ at *m/z* 1231.20 in negative mode, as well as [M+Na]^+^ at *m/z* 639.15 in positive mode.

Compound **14** was tentatively assigned as A-type dimer consisting of one (E)GC units and one (E)CG unit, which produced the pseudomolecular ion [M–H]^−^ at *m/z* 743.15 and [M+H]^+^ at *m/z* 745.15. This compound produced the fragment ion [2M+Na–2H]^−^ at *m/z* 1510.05 in negative mode while it produced the fragment ion [M+Na]^+^ at *m/z* 767.10 in positive mode.

According to the HPLC retention time and the mass information, **15** and **16** were the same compound. They were tentatively assigned as B-type dimers consisting of two (E)CG units or trimers consisting of two (E)CA and one (E)GC unit, as previously reported.[40] This compound produced the pseudomolecular ion [M+H]^+^ at *m/z* 883.15 (882.80).

Compound **17** was tentatively assigned as myricetin deoxyhexoside gallate, which produced the pseudomolecular ion [M–H]^−^ at *m/z* 615.10 and [M+Na]^+^ at *m/z* 639.50.

Compound **18** was tentatively assigned as a trimer consisting of three (E)GCG units with one A-type linkage, which produced the pseudomolecular ion [M–H]^−^ at *m/z* 1367.90 and [M+Na]^+^ at *m/z* 1391.55. Fragment ions with *m/z* 911.50 (1367.90–456.40 Da) and 455.25 (911.50–456.25) were formed in negative mode, indicating three basic (E)GCG units.

Compound **19**–**20** were tentatively assigned as dimers consisting of two (E)GCG units with one A-type linkage, which produced the pseudomolecular ion [M–H]^−^ at *m/z* 911.20 (911.25) and [M+H]^+^ at *m/z* 913.15. The two compounds were isomers.

Compound **2**, **3**, **5**, **7**, **9**, **10**, **18**, **19**, and **20** which identified by LC–MS were confirmed by MALDI-TOF MS. Yet, as reported previously[41], reversed-phase HPLC does not resolve the polymeric polyphenol extract into discrete fractions, but yields a single broad peak (Figure 5). In the RP-HPLC, proanthocyanidins are eluted according to the interaction between the mobile phase or a resin and itself. It is difficult to separate proanthocyanidin with a molecular weight exceeding that of the tetramers due to the polydispersity of proanthocyanidins[35]. Fraction 5 to 10 had compounds detected in NP-HPLC, but the polydispersity of proanthocyanidins makes them cannot be separated in RP-HPLC. Peaks were overlapped and a hump in the 280 nm RP-HPLC of each chromatograph of fraction 5 to 10 was observed. Thus, only six chromatographs were presented. Fraction 2 in NP-HPLC actually had several small peaks (right in front of peak 2). Due to the low content and low response, the small peaks were not identified in NP-HPLC-ESI/MS.

### 3.5 Antioxidant Activities

The carbohydrate content of PCBLs was 2.68±0.16 g/100 g, estimated by the anthrone-sulfuric acid method[42]. The proanthocyanidin content of PCBLs was 62.88±1.95 g/100 g, estimated by modified vanillin assay[43]. The tannin content of PCBLs was 74.46±2.39 g/100 g, estimated by the tungsten-molybdenum-phosphorus method[44]. The antioxidant activities measured by DPPH, FRAP, and ORAC assays of PCBLs were repeated three times to test the reproducibility of the assays. The EC_50_ of DPPH radical scavenging were 7.60 µg, respectively, indicating antiradical activity higher than PA extracts from peanut (0.012 mg), hazelnuts (0.015 mg), and almonds (0.015 mg), in which the extraction and purification procedure were very close [45].

Furthermore, PCBLs showed potent antioxidant activities, as evidenced by FRAP and ORAC values of 8859.33±978.39 and 12991.61±1553.34 µmol Trolox equivalents per gram of PCBLs, respectively. FRAP values of the fractions containing PAs from blackberries and peanuts are 12,925.9 µmol of catechin equivalents per gram of extract and 5859 µmol of ascorbic acid equivalents per gram of extract [45], [46]. Although the equivalents are different, the results suggest the same level of ferric-reducing capacity of PCBLs and PA extract from blackberries and peanuts. The ORAC values of fractions containing PAs from blackberries and peanuts, mangosteen oligomeric PAs, and PAs from commercially available grape seed are 3.0×10^4^, 1.47×10^4^, 1.7×10^4^, and 1.0×10^4^ µmol TE/g, respectively [45], [46], [47].

PAs in PCBLS are almost entirely of the galloylated-prodelphinidin type, which contain three –OH groups in the B ring. The presence of the functional group –OH in the structure and its position on the ring of the flavanoid molecule determine the antioxidant capacity [48]. Still, galloylated procyanidins may lead to higher antiradical activity, which support the antioxidant potential of PCBLs [49]. The degree of polymerization of PAs may also determine the antioxidant activity; higher degree of polymerization leads to higher antioxidant activity [50]. Therefore, PCBLs may be promising antioxidants.

## Conclusion

This study demonstrated by NMR spectroscopy that Chinese bayberry leaves PAs are mostly composed of (E)GCG units. The structures of proanthocyanidin polymers in Chinese bayberry leaves were first elucidated using MALDI-TOF and 95 possible structures were detected. Separating PCBLs according to their degree of polymerization by preparative normal-phase HPLC and further analysis by reversed-phase HPLC together with ESI/MS enabled comprehensive identification of oligomers. A trimer consisting of two (E)GC units and one (E)GCG unit, as well as tetrameric PAs in Chinese bayberry leaves were first identified by LC–MS. The antioxidant capacity of PCBLs was evaluated by DPPH, FRAP, and ORAC assays. The results indicate the high antioxidant potency of PCBLs, suggesting that Chinese bayberry leaves may be a source of therapeutically useful polyphenolics.
